# Case Report of Probable DRESS Syndrome Associated with Ribociclib

**DOI:** 10.1155/2023/7904950

**Published:** 2023-11-23

**Authors:** Florian Carneiro, Marine Bove, Frédérique Beau-Salinas, Tevy San, Pierre Combe

**Affiliations:** ^1^Department of Medical Oncology, University Hospital, Tours, France; ^2^Department of Medical Oncology, Hospital Center, Blois, France; ^3^Department of Pharmacosurveillance, Pharmacovigilance Regional Center, University Hospital, Tours, France; ^4^Centre d'Oncologie et Radiothérapie 37 (CORT37), Chambray les Tours, France

## Abstract

Drug reaction with eosinophilia and systemic symptoms (DRESS) is a rare but known and potentially severe side effect of drugs. The recent development of cyclin-dependent kinase 4/6 (CDK4/6) inhibitors, such as ribociclib, has considerably improved the management of hormone receptor positive (HR+) and HER2 negative (HER2-) advanced breast cancer. Here, we present the case of an 83-year-old patient who developed a probable DRESS syndrome induced by ribociclib, presenting with fever, eosinophilia, rash, and hepatic cytolysis. The RegiSCAR score was 4. The symptomatology evolved favorably with topical and systemic corticosteroids, without any sequel. Another CDK4/6 inhibitor, palbociclib, was introduced later without any cross-toxicity and with an excellent therapeutic response for more than 3 years.

## 1. Introduction

Drug reaction with eosinophilia and systemic symptoms (DRESS) syndrome is a severe delayed T-cell-mediated drug reaction. It is characterized by acute maculopapular morbilliform rash, fever, lymphadenopathy, leukocytosis with eosinophilia and atypical lymphocytes, and liver abnormalities [1, 2]. This syndrome is potentially life-threatening, with a mortality rate of 10% if not properly treated [3]. DRESS syndrome can occur from 2 to 8 weeks after introduction of the causative drug [3]. It is also known to be associated with the reactivation of herpesviruses, such as human herpesvirus 6 (HHV-6), Epstein-Barr virus (EBV), and cytomegalovirus (CMV) [4].

DRESS syndrome remains a rare side effect, with a variable incidence among different ethnicities [2]. It is usually associated with a limited number of drugs. The most common drugs responsible for DRESS syndrome are antiepileptics, in which the risk of DRESS syndrome is 2.3–4.5 in 10 000 patients [5], allopurinol, sulfasalazine, and certain antibiotics such as sulfonamides and vancomycin [3]. However, the list of potential causative agents for DRESS has considerably lengthened over the years.

The recent development of cyclin-dependent kinase 4/6 (CDK4/6) inhibitors, such as ribociclib, has improved the management of hormone receptor positive (HR+) and HER2 negative (HER2-) advanced breast cancer. The main side effects of this treatment are gastrointestinal and hematological toxicity [6]. It can also lead to some cutaneous events, such as alopecia, rash, pruritus, vitiligo-like lesions, or cutaneous lupus erythematosus [7, 8]. To our knowledge, no case of DRESS syndrome with ribociclib has been published to date.

## 2. Case Presentation

We present the case of an 83-year-old female patient diagnosed with ulcerated HR+ HER2- bilateral breast cancer with synchronous lymph node involvement and bone metastasis. Our patient had a history of hypertension and dyslipidemia treated with candesartan, bisoprolol, and atorvastatin as long-term medications. First-line treatment with letrozole 2.5 mg per day was initiated in mid-July 2019 by a gynecologist. In September 2019, the treatment was enhanced adding ribociclib and denosumab. An injection of denosumab at a dose of 120 mg was performed on 5^th^ September 2019 associated with calcium and vitamin D supplementation. Ribociclib was started on the same day at a dose of 600 mg daily but was prematurely stopped on 19^th^ September 2019 due to digestive toxicity, with grade 2 diarrhea and vomiting. Letrozole was then continued alone. During this period, the serum creatinine level increased from 0.94 mg/dL baseline to 1.38 mg/dL and 1.34 mg/dL on 9^th^ and 19^th^ September 2019, respectively. A urinary tract ultrasonography was performed, showing no evidence of obstructive uropathy nor bowel or renal structural alteration, and urine culture was sterile.

Our patient was hospitalized on 4^th^ October 2019 in the context of a fever at 39°C, grade 2 asthenia, nausea, and anorexia. Laboratory tests at admission showed hepatic cytolysis with elevation of ALT and AST at 574 IU/L and 312 IU/L, respectively. GGT and ALP were also elevated at 210 IU/L and 313 IU/L, respectively. There was no elevation of bilirubin. Eosinophilia was present, at 1460 cells/*μ*L, and was associated with presence of atypical hyperbasophilic lymphocytes. There was an acute renal failure with a creatinine level of 1.48 mg/dL, which was higher than the previously mentioned level. In the first 24 hours following her admission, she developed a pruritic maculopapular rash on her upper trunk, which extended to the arms within 24-48 hours. There was no facial edema, nor was there any recently appeared lymphadenopathy. Axillary tumoral lymphadenopathy was present but already known. A hepatic ultrasonography was performed, showing no evidence of hepatic or biliary tract structural alteration.

Viral serologies for human immunodeficiency virus (HIV), cytomegalovirus (CMV), hepatitis B virus (HBV), hepatitis C virus (HVC), hepatitis A virus (HAV), and hepatitis E virus (HEV) were negative. Serologies of treponematoses, Brucella, Coxiella, Bartonella, and Rickettsia were negative. Aspergillosis serology and dosage of mannan and galactomannan antigens were negative. The Epstein-Barr virus (EBV), measles, mumps, rubella, and toxoplasmosis serologies showed long-standing immunization. Human herpesvirus 6 (HHV6) and EBV PCRs were not performed. Hemocultures were sterile. Immunologic explorations were negative, including assays for antinuclear antibodies (ANAs), anticardiolipin antibodies (ACAs), antineutrophil cytoplasmic antibodies (ANCAs), antismooth muscle antibodies (SMAs), antimitochondrial antibodies (AMAs), anti-liver-kidney microsomal (LKM) antibodies, and liver cytosol antibody type 1 (anti-LC1).

**Figure 1:**
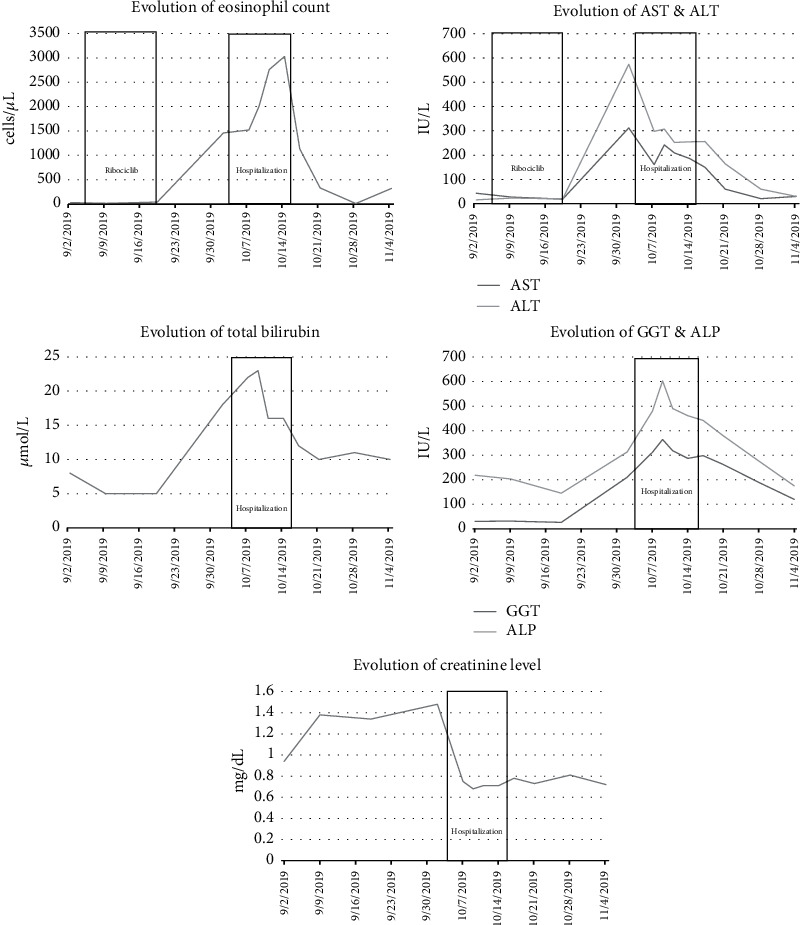
Evolution of eosinophil count, liver function tests, and creatinine level over time.

A systemic corticosteroid therapy with prednisolone at a dosage of 1 mg/kg, along with the topical application of clobetasol propionate 0.05% once daily, was initiated, followed by rapid clinical improvement. Fever and rash disappeared within three days. Hepatic cytolysis was already at its maximum at admission and normalized within 6 weeks. Total bilirubin remained subnormal, up to 23 *μ*mol/L at its maximum 15 days after hospitalization. Prothrombin ratio (PR) decreased with a minimal value of 62% 7 days after admission. Activated partial thromboplastin time (APTT) was not elevated. Eosinophilia increased to a maximum of 3030 cells/*μ*L ten days after hospitalization and then normalized within ten more days. The creatinine level quickly normalized in a few days after admission due to intravenous hydration. The evolution of biological parameters is shown in Figure 1. The RegiSCAR score was 4, favoring a probable DRESS syndrome (1 point for fever ≥ 38.5°C, atypical lymphocytes, eosinophilia ≥ 1500 cells/*μ*L, hepatic involvement, and alternative diagnoses excluded by ≥3 biological investigations; -1 point for nor edema, infiltration, purpura, or scaling).

Our patient was discharged from the hospital on 15^th^ October 2019, after 11 days of hospitalization, continuing systemic oral corticosteroids with a progressively decreasing dose. Ribociclib had already been discontinued since 19^th^ September 2019 and was never reintroduced. She initially continued letrozole alone, and another CDK4/6 inhibitor, palbociclib, was added on 16^th^ December 2019 without toxicity and is still ongoing at this day, more than 3 years later, with almost a complete metabolic response. Denosumab was also reintroduced afterward without any recurrence of toxicity to this day.

## 3. Discussion

In this situation, the clinical and biological presentation supports the diagnosis of DRESS syndrome. It is noteworthy that the symptomatology of DRESS was preceded by digestive disorders such as nausea, vomiting, and diarrhea, which led to discontinuation of the ribociclib even before the appearance of symptoms suggestive of DRESS. These digestive disorders were probably unrelated to DRESS because of their early onset and the usual absence of such manifestations in DRESS syndrome. Moreover, digestive toxicity is very common with ribociclib treatment [6].

Renal acute injury was also present, but the context of clinical dehydration linked to digestive disorders, coupled to its immediate and complete resolution after intravenous rehydration, favors functional renal insufficiency rather than visceral damage due to DRESS. Consequently, renal impairment was not included in the calculation of the RegiSCAR score.

The temporal relationship between the introduction of ribociclib, the onset of symptoms, and the clinical and biological improvement strongly suggests the causality of this molecule, even though ribociclib (which has a long elimination half-life averaging 32 hours) was discontinued 13 days before the first manifestations of DRESS.

Introduced concurrently with ribociclib, the causality of denosumab in the DRESS syndrome was subject to debate. However, we decided to attribute the causality to ribociclib and take the risk of reintroducing denosumab for two reasons. Firstly, denosumab is a well-established treatment with known adverse effects, and only one case of DRESS syndrome induced by denosumab has been reported in the literature [9]. Additionally, unlike ribociclib, for which there are equivalent therapeutic alternatives, alternatives to denosumab in the prevention of bone complications in the context of malignancies with diffuse bone involvement, such as bisphosphonates, are less effective. Not reintroducing denosumab would have been a loss for the patient. Consequently, denosumab was reintroduced, and the absence of a recurrence of toxicity is not in favor of its imputability.

To our knowledge, no cases of DRESS syndrome were revealed during clinical trials of ribociclib, and no case has been published since its marketing. However, there are four other cases, in addition to ours, of DRESS syndrome during ribociclib treatment recorded in the World Health Organization (WHO) global pharmacovigilance database, including one case that recurred after readministration. Moreover, cases of other severe cutaneous adverse reactions during ribociclib treatment have been published, such as the Stevens-Johnson syndrome and toxic epidermal necrolysis [10–13], and one case of erythema multiforme during ribociclib treatment, which reappeared after switching to palbociclib [14]. Recently, the prescribing information for KISQALI® by the Food and Drug Administration (FDA) has been updated, adding DRESS syndrome, Stevens-Johnson's syndrome, and toxic epidermal necrolysis to adverse events during postmarketing experience and warning about the risk of severe cutaneous adverse reactions during ribociclib treatment.

No case of DRESS syndrome has been published with other CDK4/6 inhibitor, such as palbociclib and abemaciclib, and there are, respectively, two cases with palbociclib and one case with abemaciclib in the WHO global pharmacovigilance database. Cases of bullous skin rashes, Stevens-Johnson's syndrome, and toxic epidermal necrolysis have been published with palbociclib [15, 16], but to our knowledge, not with abemaciclib.

## 4. Conclusion

We have presented the first published case of probable DRESS syndrome associated with ribociclib. It manifested with cutaneous and hepatic involvement and favorably responded to systemic and topical corticosteroids. It is interesting to note that treatment with another CDK4/6 inhibitor, palbociclib, could be initiated after the complete resolution of hepatic cytolysis, two months after the episode of DRESS syndrome. This treatment did not cause cross-toxicity, was very well tolerated clinically, and showed remarkable efficacy, with the persistence of an almost complete response more than 3 years after the initiation of treatment. It is important to be vigilant in monitoring patients treated with ribociclib due to the risk of severe cutaneous adverse reactions emerging since the drug's marketing.

## Data Availability

The data used to support the findings of this study are included within the article.
